# BackUp: Development and evaluation of a smart-phone application for coping with suicidal crises

**DOI:** 10.1371/journal.pone.0178144

**Published:** 2017-06-21

**Authors:** Kirsten Pauwels, Saskia Aerts, Ekke Muijzers, Eva De Jaegere, Kees van Heeringen, Gwendolyn Portzky

**Affiliations:** 1 Flemish Centre of Expertise in Suicide Prevention, Ghent, Flanders, Belgium; 2 Flemish Suicide Prevention Centre, Brussels, Brussels, Belgium; 3 Unit for Suicide Research, Ghent University, Ghent, Flanders, Belgium; Hospital Universitari de Bellvitge, SPAIN

## Abstract

**Background:**

Suicide is a major public health issue and has large impact on the lives of many people. Innovative technologies such as smartphones could create new possibilities for suicide prevention, such as helping to overcome the barriers and stigma on help seeking in case of suicidal ideation. Due to their omnipresence, smartphone apps can offer suicide prevention tools very fast, they are easily-accessible, low-threshold and can help overcome some of the help-seeking barriers suicidal people experience. This article describes the development, testing and implementation of a mobile application for coping with suicidal crisis: BackUp.

**Methods:**

Based on the analysis of literature and existing suicide prevention apps several tools were identified as relevant to include in a suicide prevention app. The selected tools (a safety planning tool, a hope box, a coping cards module, and a module to reach out) are evidence based in a face to face context, and could be easily transferred into a mobile app. The testing of existing apps and the literature also revealed important guidelines for the technical development of the application.

**Results:**

BackUp was developed and tested by an expert panel (n = 9) and a panel of end users (n = 21). Both groups rated BackUp as valuable for suicide prevention. Suicidal ideation of the end user group was measured using the Beck Scale for Suicidal Ideation before and after testing BackUp, and showed a small but non-significant decrease. The majority of the testers used BackUp several times. All tools were evaluated as rather or very useable in times of suicidal crisis.

**Conclusion:**

BackUp was positively evaluated and indicates that self-help tools can have a positive impact on suicidal ideation. Apps in particular create opportunities in approaching people that are not reached by traditional interventions; on the other hand they can contribute to suicide prevention in addition to regular care. However, more research is needed on the impact and effect of suicide prevention apps.

## Introduction

Suicide is a major public health issue and has a large impact on the lives of many people. Yearly over 800.000 people die from suicide. The global suicide rate is 15/100.000 for males, 8/100.00 for females [1]. Suicide rates in Flanders (i.e. Dutch-speaking part of Belgium) are among the highest of Western Europe, with an average rate of 23,6/100.000 for males and 10/100.000 for females [2]. The Flemish suicide rate is about 1,5 times the European average [1]. Research has indicated that, compared to neighboring countries, there appears to be a high barrier to seeking professional help in Flanders with high levels of stigma on help seeking in case of psychological distress and suicidal ideation [3].

Considering the high suicide rates and the need for low-threshold interventions, new technologies create new possibilities for suicide prevention. For several mental health problems, such as depression, new technologies including online tools and mobile applications provide new prospects in self-help, in detecting symptoms of distress, and offering direct contact to hotlines or other resources [4]. Online and mobile tools can be used in addition to traditional pharmacological and psychotherapeutic treatment, but can also be helpful for those who are reluctant or unable to find professional treatment [5]. These tools can be introduced as an additional source of support, but can also lower the barrier to seek professional help. Online tools and programs for suicide prevention create new possibilities due to their discretion, accessibility, availability and low cost, which are important barriers for help seeking [6].

Flanders has a long tradition in online suicide prevention, e.g. the suicide hotline chat and e-mail services, the digital platform Zelfmoord1813.be, which offers help and information on suicide prevention, and the app On Track Again for young suicide attempters. The reasons for developing a mobile application were numerous. Smartphones are omnipresent: 69% of Flemish inhabitants own a smartphone [7], and people have their phones with them at all times and in all places, even in time of crisis [8]. Apps can help users to detect distress and offer fast contact to a support hotline [4]. Although severe suicidal ideation requires professional treatment, many suicidal persons do not overcome help-seeking barriers such as not perceiving a need for help, access to and cost of services, fear, and stigma [8]. An app can lower the threshold to regular care and crisis lines [6, 9]. Research on the perceived usefulness of and interest in mobile applications for suicide prevention purposes stated that both clinicians, suicidal adolescents and their legal guardians endorse the use of apps [10, 11]

**Fig 1:**
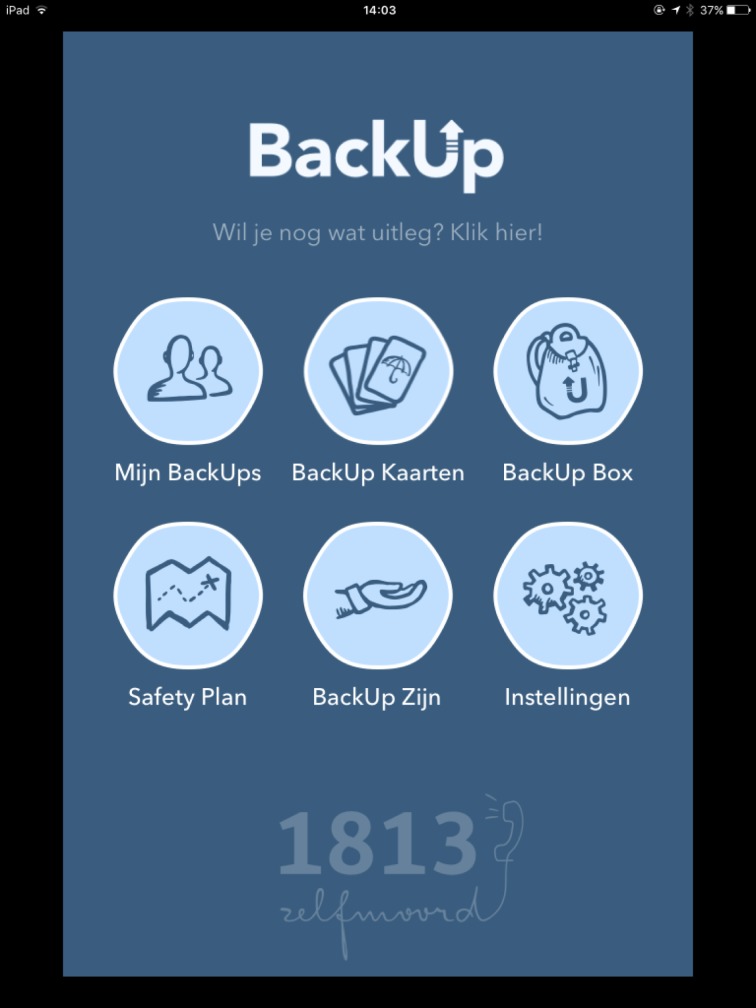
Opening screen of the BackUp app.

This article aims at describing the process of developing a mobile suicide prevention app, called BackUp (Fig 1). BackUp is developed both as a supporting tool for people who are suicidal, and as a resource for people who are concerned about a suicidal person and want to reach out. The main aims of BackUp are to provide a free, easy-accessible, independently usable application, which offers evidence-based tools to support a suicidal person while coping with a crisis. BackUp was developed by the Flemish Centre of Expertise in Suicide Prevention (VLESP), and funded by the Flemish government.

## Background research

Because the use of mobile apps in mental health care, and especially in suicide prevention, is still rather innovative, there is a lack of empirical evidence regarding the effectiveness of suicide prevention apps [10]. In addition, a solid theoretical framework is missing. However, this should not hamper the development of innovative tools that can help people in crisis. The first steps in the development of BackUp included an extensive but non-exhaustive literature search followed by a search and evaluation of available suicide prevention or mental health promoting apps.

### Literature review

We searched Web of Science for relevant studies published between 2010 and March 2017. The following key words were used: ‘suicide prevention’ or ‘mental health promotion’ in combination with ‘mobile’, ‘smartphone’, ‘app’ and ‘application’.

Apps are used for supporting online self-help courses or therapies [6, 12, 13], or to monitor the suicidal state of patients by self-reporting or registering the sleep and activity patterns of the patient [14, 15], but only a few studies specifically targeted mobile apps for suicide prevention that serve as crisis intervention tools. Christensen [16] examined research which focused on different aspects of suicide and the internet, including the effectiveness of e-health interventions. The authors stress the importance of a suicide specific approach, rather than focusing on associated symptoms of depression.

Aguirre [9] reviewed different suicide prevention applications. Most of the reviewed apps were merely providing information, or addressing other mental health problems such as depression. Few apps actually provided tools for self-use, which could be helpful for someone facing a suicidal crisis. Features which appeared to be important when developing a suicide prevention app were: the colors that are used and their potential effect on the mood of the users, providing direct access to a crisis help line, providing information on how the app content was selected and developed, and awareness of privacy issues such as how to deal with collected data. A more recent study by Luxton et al. [17] describes 14 suicide prevention apps and notes that these are the mostly used features:

- information, education and training
- resource locators and ‘emergency button’ features
- safety planning and other coping tools
- clinical assessment and automated intervention

Despite the scarce evidence on the effectiveness of mobile applications for suicide prevention, the literature review suggested that smartphone applications can offer new opportunities, especially when it comes to providing suicide prevention and crisis intervention tools that can be used when in crisis. Immediate access to crisis hotlines is presented as a key feature of mobile apps [8, 9,17]: smartphone apps can offer a very fast one touch connection to helplines or other services, also at times when a therapist is not available. Another recommended feature of suicide prevention apps is a "hope box", which has proven its utility in cognitive behavioral therapy [17, 18]. Labelle et al. [19] stress the importance of integrating a safety plan, in order to remind suicidal people of their own coping mechanisms and to offer immediate contact to their own resources (e.g. friends, family, mental health professionals).

Following the literature search, 13 apps were extensively tested: On track again, Stay Alive, Relieflink, Virtual Hope Box, Ask & Prevent Suicide, Hello Cruel World, Operation Reach Out, My3app, A friend asks, Suicide? Help!, Talk Life, Suicide Safety Plan and MoodApp. Apps that were merely informational, only focused on concerned others and/or have no interactional elements were excluded. The testing of the existing apps revealed the importance of developing a visually attractive but simple and technically-good functioning app. Additional relevant elements were features that help the users in personalizing the app according to their own preferences and needs, and that offer the possibility to add their own content (e.g. pictures, music, coping mechanisms).

### App content: Evidence based tools

Based on the literature search on suicide prevention apps and the evidence on suicide prevention in general, four self-help tools were developed for BackUp.

**Fig 2:**
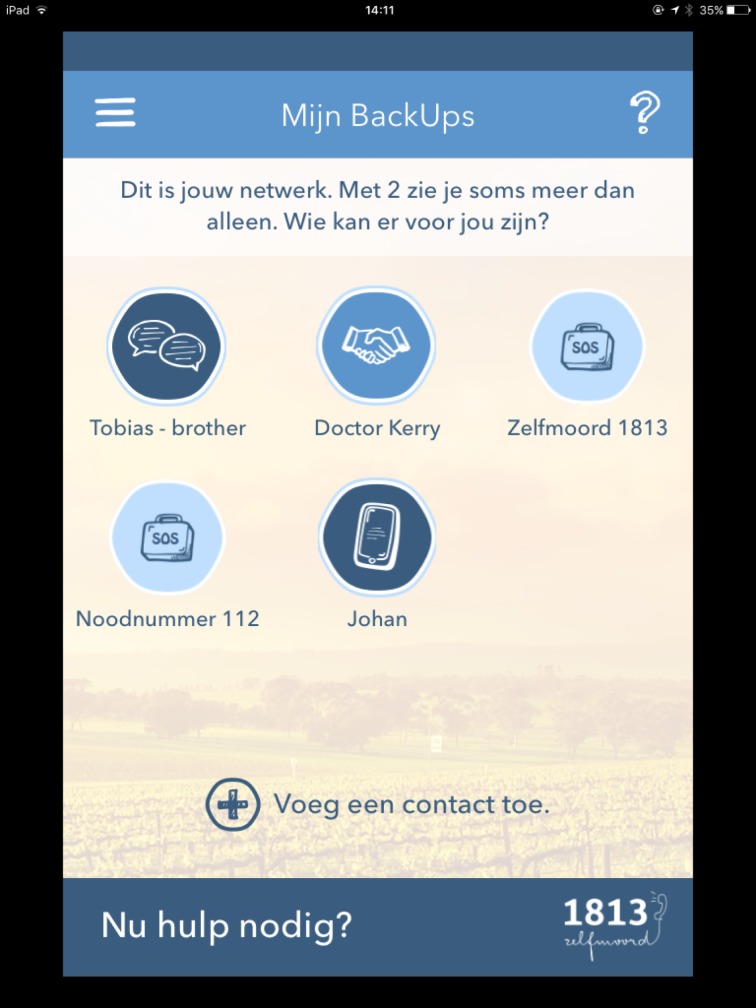
My BackUps.

**Fig 3:**
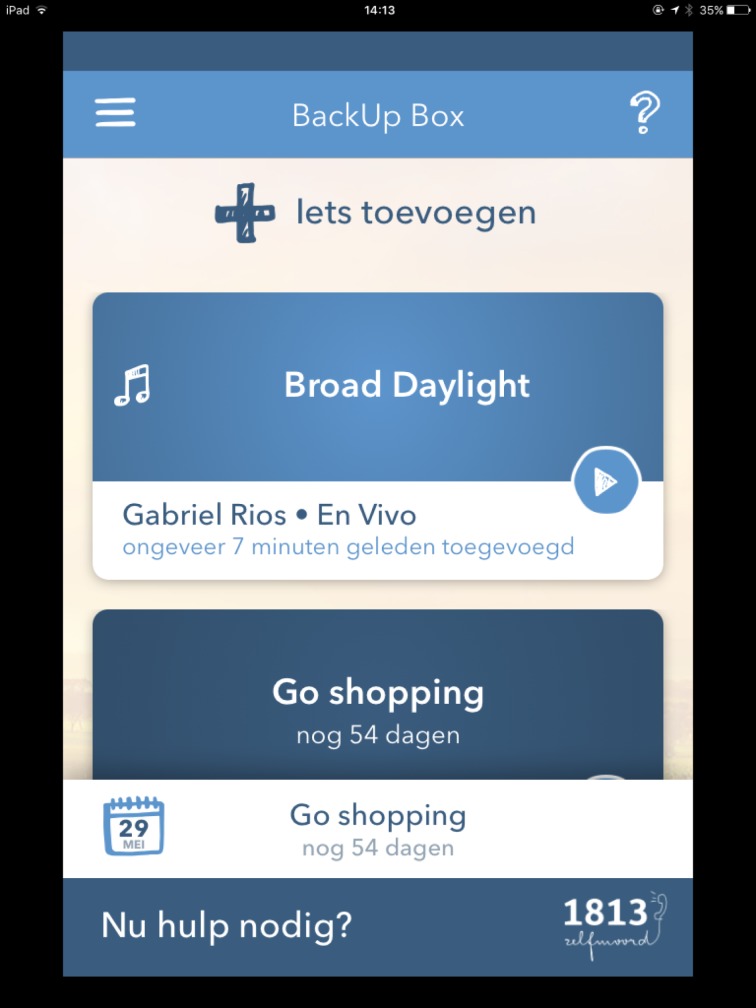
BackUp box.

**Fig 4:**
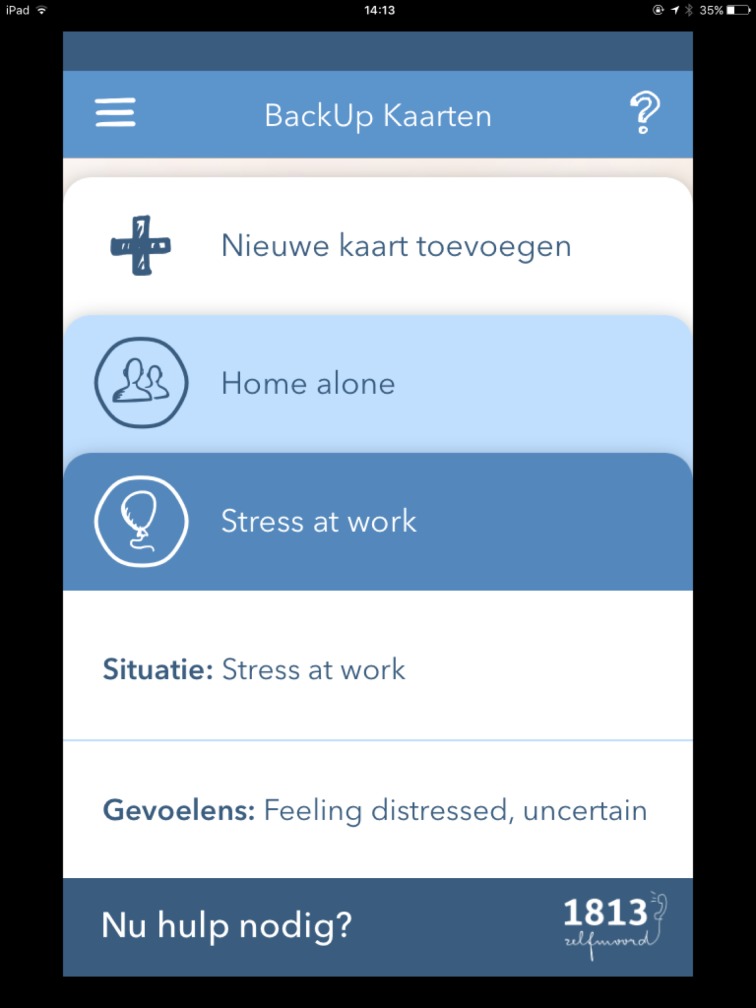
BackUp cards.

**Fig 5:**
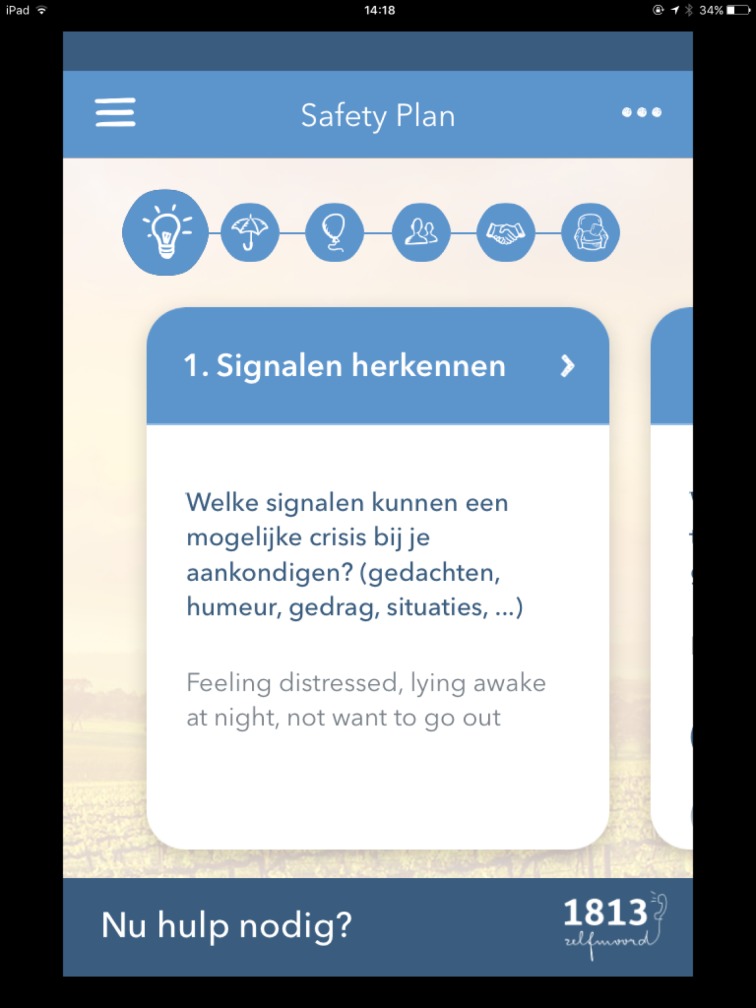
My BackUp plan.

- My BackUps (Fig 2): A tool to reach out to the person’s social network in case of suicidal crisis. Connectedness to individuals, family, community, and social institutions is described as one of the major protective factors in suicide prevention [19]. Being able to access support sources quickly and stimulating contact in times of suicidal distress can be key to suicide prevention [8].

- BackUp Box (Fig 3): This tool is based on the hope box, a tool to help suicidal people redirect their negative thoughts to reasons for living [20]. The BackUpBox offers the user a way to collect elements of hope (e.g. music, inspiring quotes or poems, pictures, memories, activities in the future) that can help to connect to reasons for living. Combating hopelessness by providing hopeful elements, a strategy that is also used in cognitive behavioral therapy, can easily be transformed in a virtual version [4].

- BackUp Cards (Fig 4): A tool that helps to identify coping strategies. This tool refers to coping cards, which are used in cognitive behavioral therapy to facilitate adaptive thinking during a suicidal crisis [20], and to manage problematic core beliefs [18]. Coping cards are more effective when they are brief, to the point and written in one’s own words [20].

- My BackUp Plan (Fig 5): A safety planning tool, based on the research of Stanley and Brown [21], which helps the user to recognize personal warning signs, to use internal coping strategies that help lower suicide risk, to reach out to family and friends who can help lower the risk, to contact appropriate professionals, and to create a safe environment.

**Fig 6:**
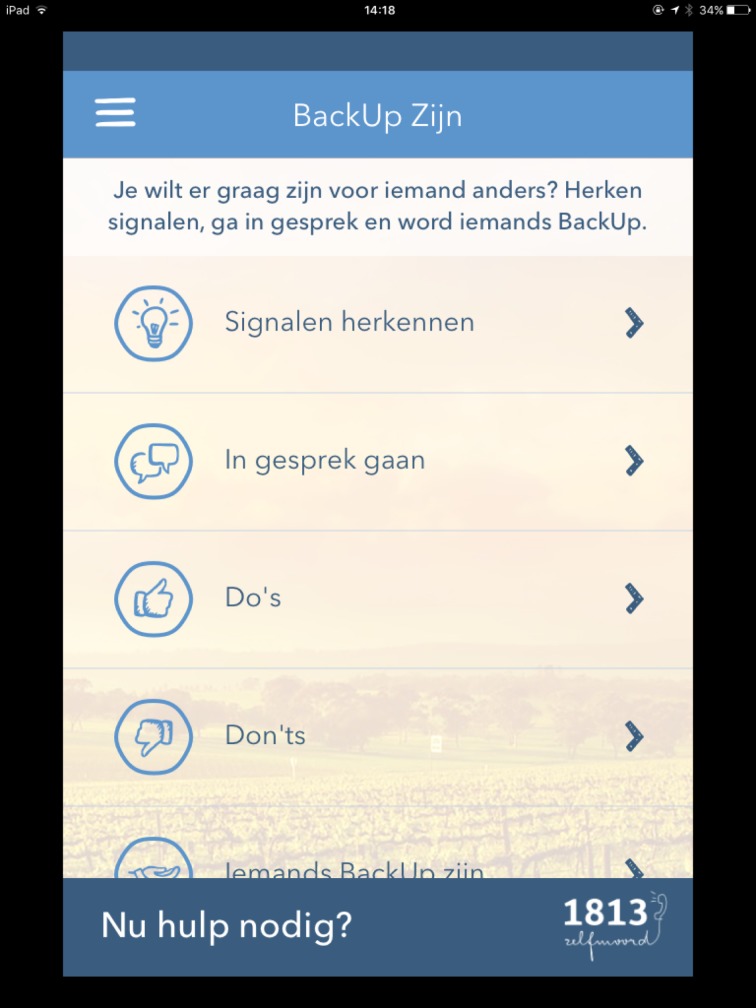
Being BackUp.

The aim of the app was not only to provide tools for suicidal persons, but also to enhance outreaching possibilities for concerned others. Reaching out and showing care to people with suicidal thoughts can have a significant, life-saving impact [22, 23]. BackUp encourages people to reach out to others by providing different connecting tools. When both a suicidal user and concerned other have installed the app, they can connect through the tool “My BackUps”. Connected users can share items from the “BackUp Box”, such as images, music or quotes that can help the suicidal person to manage a suicidal crisis. The tool “Being BackUp” (Fig 6) provides information for concerned others on detecting signals and how to talk about suicide with a suicidal person.

The app was developed in consideration of the Ethical guidelines for Technology-based Suicide prevention programmes [24]. As suggested in this guideline a permanent visibility of the suicide hotline is included in the app. On all screens users can immediately access crisis support.

To avoid a sense of defeat, which can increase suicidal behaviour [25], all competitive elements in which the user can experience the feeling of losing are avoided. Users do not have to reach a certain score and cannot fall behind on expected progress. This consideration also led to the decision not to include features like a mood barometer, follow-up schemes and diary functions. The focus of BackUp is on the here-and-now for overcoming difficult moments.

Help-seeking barriers of all kinds were avoided. Suicidal persons are very sensible to privacy issues, such as signing up for an account, no clear information about privacy policy, the collection of personal data, the use of external servers to storage data, or the use of GPS-tracking. Anonymous online tools are often preferred by people with mental health issues searching for help or information [26, 27]. BackUp collects no personal information and no external servers are used to store data. All information in BackUp is stored locally (on the user’s device), and no internet connection is needed to consult the content of the app. Another barrier for seeking help is the cost of the intervention [28], therefor BackUp is provided for free.

In the design of the app the color red was avoided because this can incite anxiety [9]. Some degree of self-customization was added, to increase user-friendliness and recognizability of the app [12]. Users can decide which opening screen they want to use, and can adjust the background picture and colours of their app.

### Using the BackUp app

BackUp is intended as an unguided self-help tool. No training or tutorial is needed to be able to use the app. Instructions are integrated in the app itself, explaining how to use the different tools. An instruction video is available on YouTube (https://www.youtube.com/watch?v=Bilke5cx5Ig) for people who need more information on how to use the app. Users can choose which tools they want to use, depending on what they prefer or feel they need the most at that moment.

However, care-givers can assist suicidal persons in using the BackUp app, e.g. helping the suicidal person with filling out the BackUp Plan of giving input for the ‘My BackUps’ tool that is integrated in the app. Instructions for care-givers on how to help somebody with the BackUp app are available online. Trainers in suicide prevention and professional care-givers are encouraged to promote using the app and to inform potential users on how to access this self-help tool.

## Results and implementation

BackUp was developed for iOS and Android, thus covering more than 95% of the Flemish smartphone market. Before launching the app, BackUp was tested in two phases: (1) a test by an independent expert team; (2) a user test by a sample of end users. Both groups evaluated the usability and the content of BackUp.

### Expert testing

A team of experts (N = 8), consisting of suicide prevention experts, psychiatrists, psychologists, and experts in online mental health, was asked to review BackUp (S1 Dataset). They were recruited from the professional network of the researchers in May 2015. They tested the application on its usability with suicidal people, but also screened it on potential adverse effects of certain content. The experts evaluated BackUp as a useable app for people with suicidal ideation, and identified all tools as valuable. Especially the safety planning tool and the BackUp Box were indicated as tools with high potential. The coping cards tool was evaluated as valuable but needed more clear instructions.

### End user testing

BackUp was tested by a sample of 21 end users (S2 Dataset). Inclusion criteria were: ≥ 18 years old, owning of a smartphone with an internet connection and operated by Android or iOS, Dutch-speaking, and having some degree of suicidal thoughts (Beck Scale for Suicidal Ideation (BSS) ≥ 1). The sample consisted of 16 women and 5 men, with an average age of 30 years old (age range: 18–54). The study was approved by the ethical committee of the Ghent University hospital and all the participants agreed to an online informed consent before taking part in the research. Participants were recruited in June 2015 through the Flemish suicide prevention portal www.zelfmoord1813.be and online advertising on Facebook. The testers were asked to use BackUp during one week (with no further instructions about the frequency of use), and their level of suicidal ideation was registered with the BSS [29] both before and after the testing period. The average BSS-score of the participants at baseline was 18.5. Nine participants had a BSS-score higher than or equal to 26. For those participants a safety procedure was installed. The safety procedure consisted of a telephone call by a staff member of the suicide prevention center in which their suicide risk was assessed. All participants received a referral card (both at baseline and after the testing period) with contact details of mental health care institutions.

Of the forty-five candidates who registered for the study, twenty-one participants filled in both the pre- and posttest. The average BSS of this group was 20.1 at baseline and 18.7 at posttest, which indicates a small but non-significant decrease after one week using BackUp. The drop-out percentage of the participants with high BSS was low (33%) compared to the average drop-out rate of the testing group (54%). Next to the BSS-measures, the participants were asked to evaluate BackUp and to comment on the support they experienced from the application. They were asked to rate the different tools and indicate which ones they preferred.

**Fig 7:**
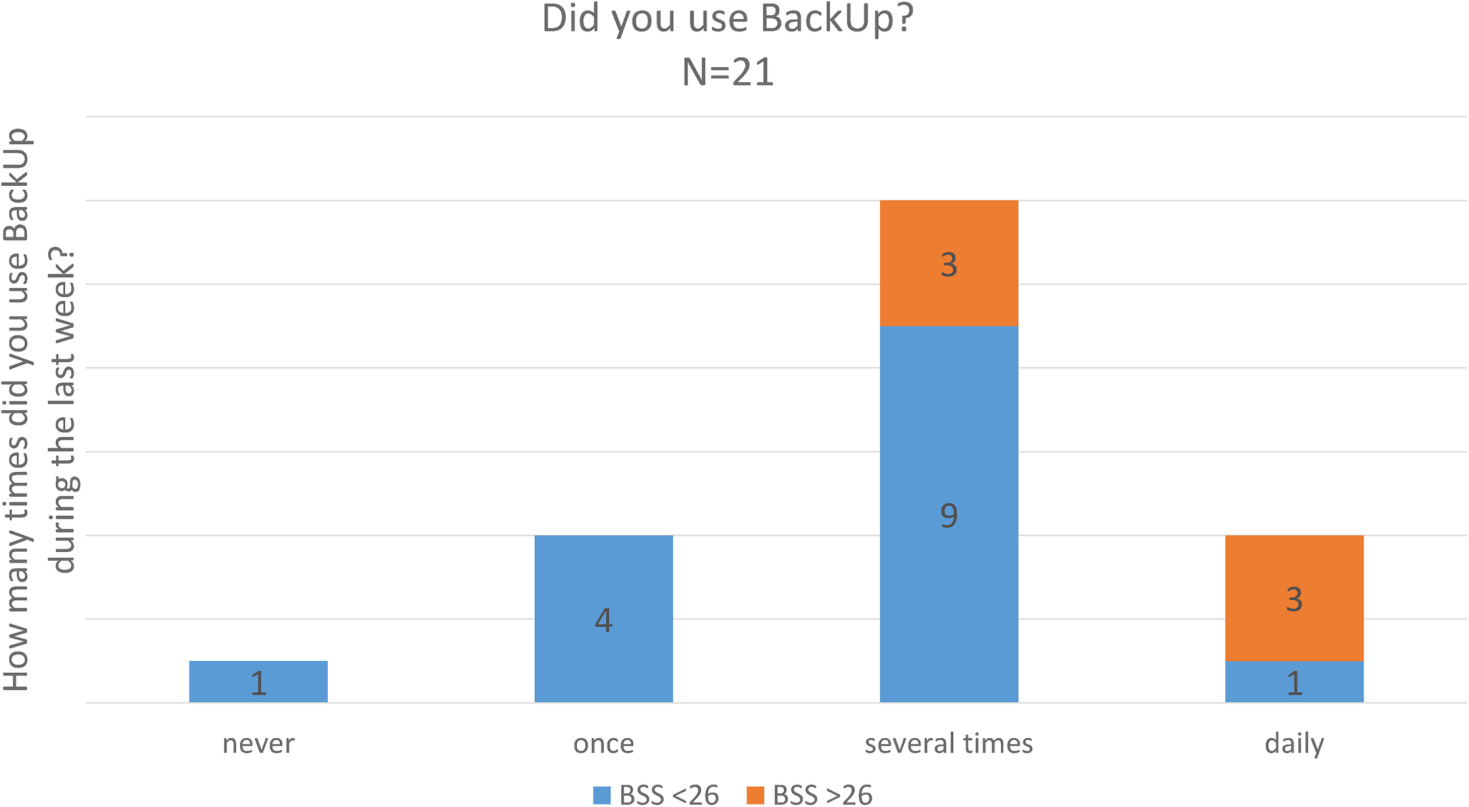
Usage of BackUp during testing period.

Of the test sample twenty participants (95%) used BackUp at least once during the testing period, sixteen used the application several times (Fig 7). All high suicidal testers (N = 6) used the application more than once. Four (20%) testers do not think BackUp can help them cope with suicidal thoughts. When asked if they would use the app in their daily life, fourteen (70%) participants agreed.

**Fig 8:**
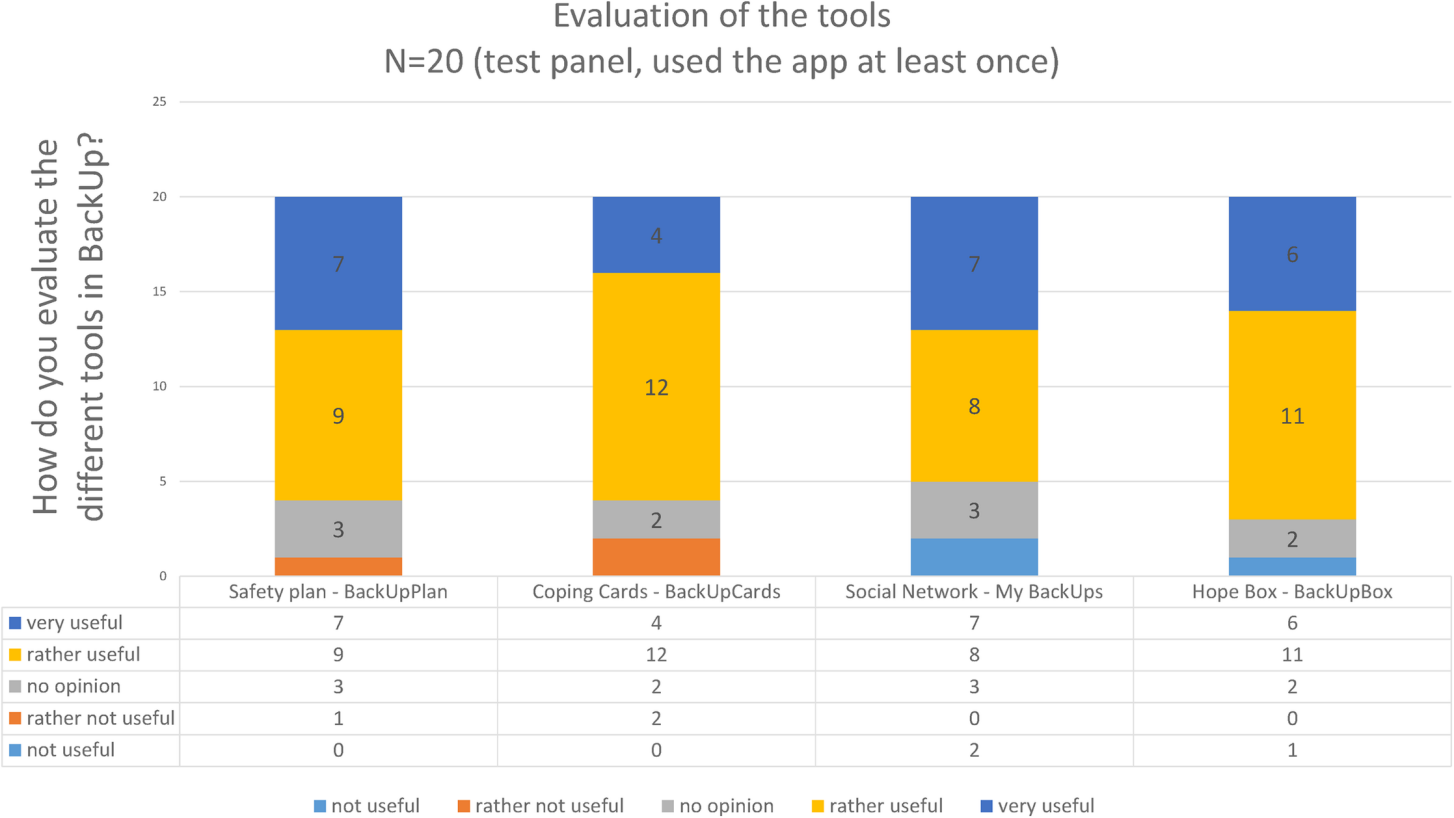
Evaluation of the tools by test panel.

All four self-help tools were evaluated as (rather or very) useable when suicidal (N = 20). All testers at least evaluated one item as very useable (Fig 8).

### Launch of BackUp

BackUp was launched in September 2015. The first six months, BackUp was downloaded nearly 1.500 times. A user survey is implemented in the app, and is offered to all users one month after downloading BackUp. The results of this survey should contribute to further improvement of BackUp.

At the beginning of 2017, a first update of the BackUp app was introduced. This update included some technical improvements, and based on the user survey some of the tools were made even more personalizable, e.g. users can import own pictures as background picture and can add own warning signs and do’s and don’ts to the “Being BackUp” tool.

## Conclusions

The available literature indicates that a mobile suicide prevention app can be a valuable addition to existing mental health resources and can be especially useful for suicidal persons who may otherwise not seek (professional) help. It may be of great value to many and has a high potential to reach its goal: providing suicidal persons with tools to keep themselves safe, especially during a suicidal crisis.

The development of BackUp was inspired by a combination of knowledge of evidence-based suicide prevention strategies and the new possibilities of mobile phone technology. The available evidence regarding the effectiveness of suicide prevention apps in reducing suicidal behaviour was limited, so the development was guided by results and recommendations from research on other online and offline self-help tools and strategies [4, 9]

BackUp was well received by both suicide prevention experts and the target audience. The user test indicated that using the app could help suicidal persons in dealing with suicidal thoughts and coping with an upcoming suicidal crisis. These findings, combined with more recent studies on the effectiveness of other mobile applications in suicide prevention [8], show that the self-help tools that were included in BackUp can have a positive impact on reducing suicidal thoughts.

More research on the effectiveness of suicide prevention apps is needed, but integrating mobile technology in suicide prevention appears to be promising. Apps have the advantage to reach many people since they are easily accessible. A well-designed and easy to use app, based on effective suicide prevention strategies, can be a welcome addition to regular mental health care. It can help persons in need to cope with a suicidal crisis by providing hope and the possibility to reach out for help. Via suicide prevention apps, suicidal people who are not reached by regular mental health care because of financial, spatial, or other help seeking barriers, can get help. Moreover, BackUp can also be used in addition to regular care. BackUp is designed in such a way that suicidal persons can use it on their own, but also together with a caregiver and/or concerned other.

## Supporting information

S1 Dataset Expert testing data. (XLSX)

S2 Dataset End user testing data. (XLSX)
